# Supplementary material for: Identification and functional analysis of non-coding regulatory small RNA FenSr3 in Bacillus amyloliquefaciens LPB-18
Source: PeerJ. 2023 May 15;11:e15236. doi: 10.7717/peerj.15236 (PMC10194069; doi:10.7717/peerj.15236)
Supplement: Supplemental Information 4 [file peerj-11-15236-s004.zip › KO/CK-vs-T1_map/map05225.html]

KEGG PATHWAY: Hepatocellular carcinoma - Reference pathway


|  |  |
| --- | --- |
| **Hepatocellular carcinoma - Reference pathway** |  |

[
Pathway menu
| Organism menu
| Pathway entry
| Show description
| User data mapping
]

Hepatocellular carcinoma (HCC) is a major type of primary liver cancer and one of the rare human neoplasms etiologically linked to viral factors. It has been shown that, after HBV/HCV infection and alcohol or aflatoxin B1 exposure, genetic and epigenetic changes occur. The recurrent mutated genes were found to be highly enriched in multiple key driver signaling processes, including telomere maintenance, TP53, cell cycle regulation, the Wnt/beta-catenin pathway (CTNNB1 and AXIN1), the phosphatidylinositol-3 kinase (PI3K)/AKT/mammalian target of rapamycin (mTOR) pathway. Recent studies using whole-exome sequencing have revealed recurrent mutations in new driver genes involved in the chromatin remodelling (ARID1A and ARID2) and the oxidative stress (NFE2L2) pathways.

|  |  |  |
| --- | --- | --- |
| Reference pathway | 184% 150% 122% 100% 82% 67% 55% | 图片下载 |
